# Prevalence, trend, and predictor analyses of vitamin D deficiency in the US population, 2001–2018

**DOI:** 10.3389/fnut.2022.965376

**Published:** 2022-10-03

**Authors:** Aiyong Cui, Peilun Xiao, Yuzhuo Ma, Zhiqiang Fan, Fengjin Zhou, Jiang Zheng, Liang Zhang

**Affiliations:** ^1^Department of Orthopedics, Honghui Hospital, Xi'an Jiaotong University, Xi'an, China; ^2^Department of Orthopaedics, The Fifth Affiliated Hospital of Sun Yat-sen University, Zhuhai, Guangdong, China; ^3^Department of Pelvic and Acetabular Surgery, Honghui Hospital, Xi'an Jiaotong University, Xi'an, China; ^4^Department of Sports Medicine, Honghui Hospital, Xi'an Jiaotong University, Xi'an, China

**Keywords:** prevalence, trends, vitamin D, predictors, NHANES

## Abstract

**Background:**

The National Health and Nutrition Examination Surveys (NHANES) collect and release data to the public every 2 years. The latest NHANES study on the vitamin D status of Americans was based on data from 2001 to 2014, and the latest data (2015–2016 and 2017–2018) have not been studied yet. Thus, we extracted all the available data from NHANES (2001–2018), aiming to analyze the prevalence and trends of vitamin D deficiency (VDD) in the US population to bridge the research gap.

**Methods:**

According to previous studies and nutritional guidelines for vitamin D, severe VDD was defined as serum 25(OH)D levels of <25 nmol/L, moderate deficiency as 25–50 nmol/L, insufficiency as 50–75 nmol/L, and sufficiency as >75 nmol/L. We comprehensively estimated the prevalence of serum 25(OH)D levels of <25, 25–50, 50–75, and >75 nmol/L in Americans and described trends in vitamin D status from 2001 to 2018. Weighted multivariate linear regression models were used to explore the predictors of VDD. All analyses and the data were adjusted for the complex sampling design of NHANES using Mobile Examination Center (MEC) weights.

**Results:**

Based on the most recent data of 71,685 participants, our study showed that the weighted prevalence of severe and moderate VDD was 2.6% and 22.0%, and the prevalence of vitamin D insufficiency (VDI) and sufficiency was 40.9% and 34.5%. The prevalence of severe and moderate VDD was higher in women, non-Hispanic black Americans, people aged 20–29 years, and during the season of winter. From 2001 to 2018, we found a slight linear decrease in the prevalence of moderate VDD (coefficient = −0.847; *P* = 0.009) and VDI (coefficient = −0.810; *P* = 0.014). We also found a slight linear increase in vitamin D sufficient (coefficient = 1.693; *P* = 0.004). However, no trend change was observed in severe VDD (coefficient = −0.037; *P* = 0.698). Age, sex, ethnicity, season, sun-protective behaviors, lower BMI, lower socioeconomic status (SES), drinking, and lower milk consumption were predictors of severe VDD.

**Conclusion:**

Vitamin D deficiency is still prevalent in the United States, especially in non-Hispanic black Americans, women, individuals aged 20–29, and during winter. Therefore, individuals, healthcare providers, and policymakers should take public health measures to develop and implement prevention strategies to deal with VDD.

## Introduction

Vitamin D plays a critical role in maintaining bone mineralization by influencing calcium and phosphorus homeostasis (1). Vitamin D deficiency (VDD) is associated with an increased risk of rickets and osteomalacia (2, 3). Evidence has shown that VDD is associated with extraskeletal conditions, such as infection, cancer, diabetes mellitus, cardiovascular disease, and autoimmune disease (4–7). Recently, Pugach et al. (8) reported a strong association between vitamin D status and the mortality rate from COVID-19 in Europe. Vitamin D deficiency has become a global health issue (9), which is usually linked to insufficient exposure to sunlight and insufficient consumption of vitamin D-rich foods (10). Ultraviolet-B (UVB) radiation (wavelength 290–315 nm) in the sun rays could penetrate the skin and convert 7-dehydrocholesterol to pre-vitamin D3 and then vitamin D3 (11). *In vivo*, vitamin D absorbed from food is hydroxylated in the liver to 25-hydroxyvitamin D, then in the kidneys to 1,25-dihydroxyvitamin D to play a fundamental role in bone metabolism or other physiological processes. The Institute of Medicine (IOM) recommended 600 IU/d vitamin D intake for ages 1–70 years and 800 IU/d for ages 71 years and older (12). A recent study showed that the prevalence of VDD was still high in sunny Africa, and 32.2% of Africans were estimated to have VDD when a cut-off of serum 25(OH)D <50 nmol/L was used (13). In the United States, VDD is also a growing public health concern (14). However, due to the controversial definition of VDD, the results of different studies varied widely (12, 15).

Vitamin D deficiency has been defined differently depending on the effects on parathyroid hormone suppression, maximum calcium absorption, or bone mineral density (BMD) (16). Institute of Medicine defined people at risk of VDD as serum 25(OH)D <30 nmol/L (12 ng/L) and at risk of insufficiency as 30 <25(OH)D <50 nmol/L (12–20 ng/L) (12). However, the Endocrine Society (ES) suggested VDD as a serum 25(OH)D <50 nmol/L and vitamin D insufficiency (VDI) as 50 <25(OH)D <75 nmol/L (17). Several National Health and Nutrition Examination Survey (NHANES) studies have estimated the vitamin D status of Americans by different cut-offs. National Health and Nutrition Examination Survey (2000–2004) reported that 5% of Americans had 25(OH)D levels <27.5 nmol/L (15). Liu et al. (18) estimated that 28.9% and 41.4% of American adults had VDD [25(OH)D <50 nmol/l] and VDI [50 ≤ 25(OH)D <75 nmol/l] using NHANES 2001–2011. In this study, the prevalence was much higher among the elderly, non-Hispanic black Americans, people with obesity, and people who reported little milk consumption. Another study by Herrick et al. (19) investigated the vitamin D status in America using NHANES 2011–2014. They found the prevalence of 25(OH)D levels <30 and 30–50 nmol/L was 5.0% and 18.3%, respectively.

In August 2021 and April 2022, the NHANES website released the data on vitamin D from 2015 to 2016 and 2017 to 2018. However, there has not been a study analyzing these data. Therefore, this study aims to examine the American population's vitamin D status using currently available data from NHANES (2001–2018). This report also provides the trends for VDD and VDI in the United States from 2001 to 2018.

## Methods

### Study design and population

National Health and Nutrition Examination Survey is a nationally representative nutrition survey of general populations in the United States using a stratified, multi-stage random sampling design. As the latest data for vitamin D status are available as of NHANES 2017–2018, nine consecutive cycles of NHANES (2001–2002, 2003–2004, 2005–2006, 2007–2008, 2009–2010, 2011–2012, 2013–2014, 2015–2016, and 2017–2018) were selected for our analysis. Serum 25(OH)D data were available for subjects older than 6 years in 2001–2002 and older than 1 year in 2003–2018. We initially included 91,351 subjects from NHANES 2001–2018. After excluding 19,666 subjects without serum 25(OH)D data, 71,685 eligible subjects were included in this analysis. 25(OH)D concentrations were measured in the south during the winter months (November–March) and in the north during the summer months (April–October). The total serum 25(OH)D concentration was defined as the sum of 25(OH)D2 and 25 (OH)D3. Serum 25(OH)D concentrations were measured at the National Center for Environmental Health using the DiaSorin RIA kit (Stillwater MN) in 2001–2006 and the standardized liquid chromatography-tandem mass spectrometry (LC-MS/MS) method in 2007–2018. Considering the quality control issues of the RIA in NHANES 2001–2006, the Centers for Disease Control and Prevention (CDC) decided to adjust and convert the measured values of 25(OH)D concentrations to equivalent measurements by LC-MS/MS methods. It allows us to combine and compare these data. The detailed method for adjustment can be found on the NHANES website and in Appendix 1 in Supplementary material.

Since the definition of VDD was still controversial, we defined severe VDD as serum 25(OH)D levels of <25 nmol/L, moderate deficiency as 25–50 nmol/L, insufficiency as 50–75 nmol/L, and sufficient as >75 nmol/L according to previous studies (12, 17, 20).

### Potential predictors of vitamin D deficiency

Based on previous studies, we selected factors that may contribute to VDD (18, 21). Participants were divided into four groups based on their physiological characteristics: <18, 18–44, 45–65, and >65 years old. Race/ethnicity was classified as non-Hispanic white, non-Hispanic black Americans, Mexican Americans, other Hispanics, and other races. Body mass index (BMI) was classified into three categories: BMI <18.5, 18.5–25, and >25. The education level was divided into two groups: high-school degree and below, college degree and above. The poverty income ratio (PIR) was calculated by dividing family income by the poverty threshold in the survey year. Many studies have used it as the primary indicator of socioeconomic status (SES) (22, 23). The PIR was divided into three categories: low-income (PIR ≤ 1.3), middle-income (PIR > 1.3–3.5), and high-income (PIR > 3.5) based on previous literature (24). Based on a previous study, we also discussed the effect of sun-protective behaviors on VDD (25). We collected three sun-protective behaviors from three questionnaires—staying in the shade, wearing long-sleeved shirts, and using sunscreen. Responses for all three behaviors included “always,” “most of the time,” “sometimes,” “rarely,” and “never.” We defined sun-protective behaviors as frequent (always or most of the time), moderate (sometimes), or rare (never or rarely). Physical activity was collected through a questionnaire—how many days in the past 7 days did you perform a total of at least 60 min of exercise? We defined physical activity as rare (0 days), sometimes (1–3 days), and regular (4–7 days). Smoking and drinking behaviors were determined by a questionnaire: Have you smoked at least 100 cigarettes in your life? Have you had at least 12 alcoholic drinks in 1 year? Milk consumption was determined by a questionnaire: Have you had regular milk use five times per week with three answers, namely, “I had never been a regular milk drinker,” “I sometimes was a regular milk drinker in my life,” and “I have been a regular milk drinker for most or all of life.” Individuals with incomplete data (Label description as refused, don't know, and missing) were pre-excluded when weighted linear regression analyses were performed. Details on the above data can be found on the NHANES website (http://www.cdc.gov/nchs/nhanes/).

### Statistical analysis

All analyses and data were adjusted for the complex sampling design of NHANES using Mobile Examination Center (MEC) weights. We used percentages for categorical variables and means ± standard deviations for continuous variables. To compare the difference between the groups, we used the weighted χ^2^-test and the Wald *F*-test for categorical and continuous variables, respectively. We calculated the weighted prevalence of serum 25(OH)D levels <25, 25–50, 50–75, and >75 nmol/L by dividing the weighted number of people with different vitamin D concentrations by the weighted total number of people in the study. From 2001 to 2018, linear trend tests were used to identify trends in vitamin D status in Americans. A *P*-value of <0.05 was considered statistically significant.

Multivariate linear regression models were used to explore the predictors of serum 25(OH)D levels <25 and 50 nmol/L, controlling for a large range of variables. The following predictors were explored, including age group (<18, 18–44, 45–65, and >65 years old), sex (female and male), race/ethnicity (non-Hispanic whites, non-Hispanic blacks, Mexican Americans, other Hispanics, non-Hispanic blacks, other races), education (high-school degree and below, college degree and above), PIR (<1.3, 1.3–3.5, and >3.5), BMI (<18.5, 18.5–25, and >25 kg/m^2^), season (winter and summer), sun-protective behaviors, smoking behaviors, alcohol consumption, physical activity, and milk consumption. A *P*-value of <0.05 was considered statistically significant. All statistics were performed using the R software (version 4.2.0) and EmpowerStats (http://www.empowerstats.com).

## Results

Overall, 71,685 subjects were included in our analysis, with a mean age of 34.8 ± 24.0. In this study, 20.7% of the participants were Mexican Americans, 8.4% were other Hispanics, 38.2% were non-Hispanic whites, 23.0% were non-Hispanic blacks, and 9.7% were other races (including multiracial). 48.1% of the tests were carried out in winter, and 51.9% in summer. The characteristics of the participants are listed in Table 1.

**Table 1:** Descriptive characteristics of the participants (*n* = 71,685) in the present study.

**Characteristic**	**%**	**95 % CI**
**Sex**		
Female	50.4	(50.3–51.1)
**Race/Ethnicity**		
Mexican American	20.7	(20.4–21.0)
Other Hispanics	8.4	(8.2–8.6)
Non-Hispanic white	38.2	(37.8–38.6)
Non-Hispanic black	23.0	(22.7–23.3)
Other race	9.7	(9.5–9.9)
**Age**		
<18	34.1	(33.8–34.5)
18–44	31.0	(30.7–31.4)
45–65	19.9	(49.8–50.9)
>65	15.0	(14.7–15.3)
**Education**		
High school degree and below	49.7	(49.2–50.2)
College degree and above	50.3	(49.8–50.9)
**PIR**		
<1.3	34.3	(33.9–34.7)
1.3–3.5	41.1	(40.8–41.5)
>3.5	24.6	(24.2–24.9)
**Sun-Protective Behaviors**		
**Staying in the shade**		
Rare	35.1	(34.5–35.8)
Sometimes	38.6	(37.9–39.3)
Frequent	26.3	(25.7–26.9)
**Wearing long sleeves**		
Rare	10.8	(10.3–11.2)
Sometimes	19.7	(19.2–20.3)
Frequent	69.5	(68.9–70.3)
**Using sunscreen**		
Rare	22.9	(22.3–23.5)
Sometimes	19.1	(18.5–19.6)
Frequent	58.0	(57.4–58.6)
**Season**		
Winter	48.1	(47.1–49.0)
Summer	51.9	(50.0–53.0)
**Had at least 12 alcohol drinks/1 year?**		
Yes	70.1	(69.6–70.5)
No	29.9	(29.5–30.4)
**Smoke at least 100 cigarettes in life**		
Yes	45.9	(45.4–46.4)
No	54.1	(53.6–54.6)
**Physical activity**		
Rare	4.5	(4.0–5.1)
Sometimes	15.1	(14.2–16.0)
Regular	80.4	(79.4–81.4)
**BMI (vs**. ** <18.5)**		
<18.5	15.9	(15.6–16.2)
18.5–25	31.0	(30.6–31.3)
>25	53.1	(52.7–53.5)
**Milk consumption (vs. Rare)**		
Rare	22.9	(22.5–23.3)
Sometimes	35.1	(34.6–35.5)
Regular	42.0	(41.6–42.5)

PIR, poverty income ratio; BMI, body mass index.

The weighted prevalence of serum 25(OH)D levels <25, 25–50, 50–75, and >75 nmol/L was 2.6% (95% CI 2.5–2.7), 22.0% (95% CI 21.7–22.3), 40.9% (95% CI 40.5–41.3), and 34.5% (95% CI 34.2–34.9) in Americans older than 1 year, respectively (Table 2).

**Table 2:** Weighted prevalence of serum 25(OH)D <25, 25–50, 50–75, and >75 nmol/l by gender, ethnicity, age, and season.

	**Sample size**	** <25 nmol/L**	**95% CI**	**25–50 nmol/L**	**95 CI**	**50–75 nmol/L**	**95% CI**	**>75 nmol/L**	**95 CI**	***P***
All	71,685	2.6%	(2.5%, 2.7%)	22.0%	(21.7%, 22.3%)	40.9%	(40.5%, 41.3%)	34.5%	(34.2%, 34.9%)	
Gender										<0.001
Male	35,306	2.1%	(2.0%, 2.3%)	21.2%	(20.8%, 21.6%)	44.4%	(43.9%, 44.9%)	32.3%	(31.8%, 32.8%)	
Female	36,379	3.1%	(2.9%, 3.3%)	22.8%	(22.4%, 23.2%)	37.5%	(37.0%, 38.0%)	36.6%	(36.1%, 37.1%)	
Ethnicity										<0.001
Mexican American	14,822	3.2%	(2.9%, 3.5%)	35.1%	(34.3%, 35.9%)	47.5%	(46.7%, 48.3%)	14.2%	(13.7%, 14.8%)	
Other Hispanics	6,017	2.3%	(1.9%, 2.7%)	28.7%	(27.6%, 29.9%)	49.4%	(48.1%, 50.7%)	19.6%	(18.6%, 20.6%)	
Non-Hispanic white	27,411	0.9%	(0.8%, 1.0%)	13.8%	(13.4%, 14.2%)	41.1%	(40.5%, 41.7%)	44.2%	(43.6%, 44.8%)	
Non-Hispanic black	16,467	11.9%	(11.4%, 12.4%)	48.5%	(47.7%, 49.3%)	28.9%	(28.2%, 29.6%)	10.7%	(10.2%, 11.2%)	
Other race—including multi-racial	6,968	3.2%	(2.8%, 3.6%)	31.2%	(30.1%, 32.3%)	41.3%	(40.2%, 42.5%)	24.3%	(23.3%, 25.3%)	
Males—age (years)										<0.001
1–9	5,843	0.3%	(0.2%, 0.5%)	8.0%	(7.3%, 8.7%)	47.4%	(46.1%, 48.7%)	44.3%	(43.0%, 45.6%)	
10–19	8,000	1.7%	(1.4%, 2.0%)	20.0%	(19.7%, 21.5%)	50.2%	(49.1%, 51.3%)	27.5%	(26.5%, 28.5%)	
20–29	3,546	3.5%	(2.9%, 4.2%)	29.2%	(27.7%, 30.7%)	44.5%	(42.9%, 46.1%)	22.8%	(21.4%, 24.2%)	
30–39	3,500	2.9%	(2.4%, 3.5%)	27.0%	(25.6%, 28.5%)	45.3%	(43.7%, 47.0%)	24.8%	(23.4%, 26.3%)	
40–49	3,564	2.2%	(1.8%, 2.7%)	22.4%	(21.1%, 23.8%)	44.7%	(43.1%, 46.3%)	30.7%	(29.2%, 32.2%)	
50–59	3,356	2.3%	(1.8%, 2.9%)	21.2%	(19.8%, 22.6%)	41.5%	(39.9%, 43.2%)	35.0%	(33.4%, 36.6%)	
60–69	3,600	1.5%	(1.2%, 2.0%)	17.7%	(16.5%, 19.0%)	40.5%	(38.9%, 42.1%)	40.3%	(38.7%, 41.9%)	
70–79	2,427	1.3%	(0.9%, 1.9%)	15.4%	(14.0%, 16.9%)	38.6%	(36.7%, 40.6%)	44.7%	(42.7%, 46.7%)	
>80	1,470	2.1%	(1.6%, 3.1%)	16.8%	(15.0%, 18.8%)	36.1%	(33.7%, 38.6%)	45.0%	(42.5%, 47.6%)	
*P*										
Females—age (years)										<0.001
1–9	5,617	0.3%	(0.2%, 0.5%)	10.1%	(9.3%, 10.9%)	48.3%	(47.0%, 49.6%)	41.3%	(40.0%, 42.6%)	
10–19	7,764	3.0%	(2.6%, 3.4%)	26.7%	(25.7%, 27.7%)	44.8%	(43.7%, 45.9%)	25.5%	(24.5%, 26.5%)	
20–29	4,106	4.7%	(4.1%, 5.4%)	27.8%	(26.4%, 29.2%)	36.3%	(34.8%, 37.8%)	31.2%	(29.8%, 32.6%)	
30–39	3,944	3.6%	(3.1%, 4.2%)	26.3%	(24.9%, 27.7%)	38.8%	(37.3%, 40.3%)	31.3%	(29.9%, 32.8%)	
40–49	3,943	4.0%	(3.4%, 4.7%)	24.7%	(23.4%, 26.1%)	38.1%	(36.6%, 39.6%)	33.2%	(31.8%, 34.7%)	
50–59	3,415	3.1%	(2.6%, 3.7%)	21.6%	(20.3%, 23.0%)	35.5%	(33.9%, 37.1%)	39.8%	(38.2%, 41.5%)	
60–69	3,656	2.3%	(1.9%, 2.8%)	20.7%	(19.4%, 22.1%)	29.5%	(28.1%, 31.0%)	47.5%	(45.9%, 49.1%)	
70–79	2,317	2.7%	(2.1%, 3.5%)	17.9%	(16.4%, 19.5%)	29.3%	(27.5%, 31.2%)	50.1%	(48.1%, 52.1%)	
>80	1,617	2.4%	(1.8%, 3.3%)	17.2%	(15.4%, 19.1%)	29.0%	(26.8%, 31.3%)	51.4%	(49.0%, 53.8%)	
Season										<0.001
Winter	34,467	4.1%	(3.9%, 4.3%)	28.9%	(28.4%, 29.4%)	40.7%	(40.2%, 41.2%)	26.3%	(25.8%, 26.8%)	
Summer	37,218	1.6%	(1.5%, 1.7%)	17.0%	(16.6%, 17.4%)	41.0%	(40.5%, 41.5%)	40.5%	(40.0%, 41.0%)	

The weighted χ^2^-test was used for categorical variables, and the Wald F-test was used for continuous variables to compare the difference between groups. P-value <0.05 was considered statistically significant. MEC weights were used. MEC, Mobile Examination Center.

**Figure 1:**
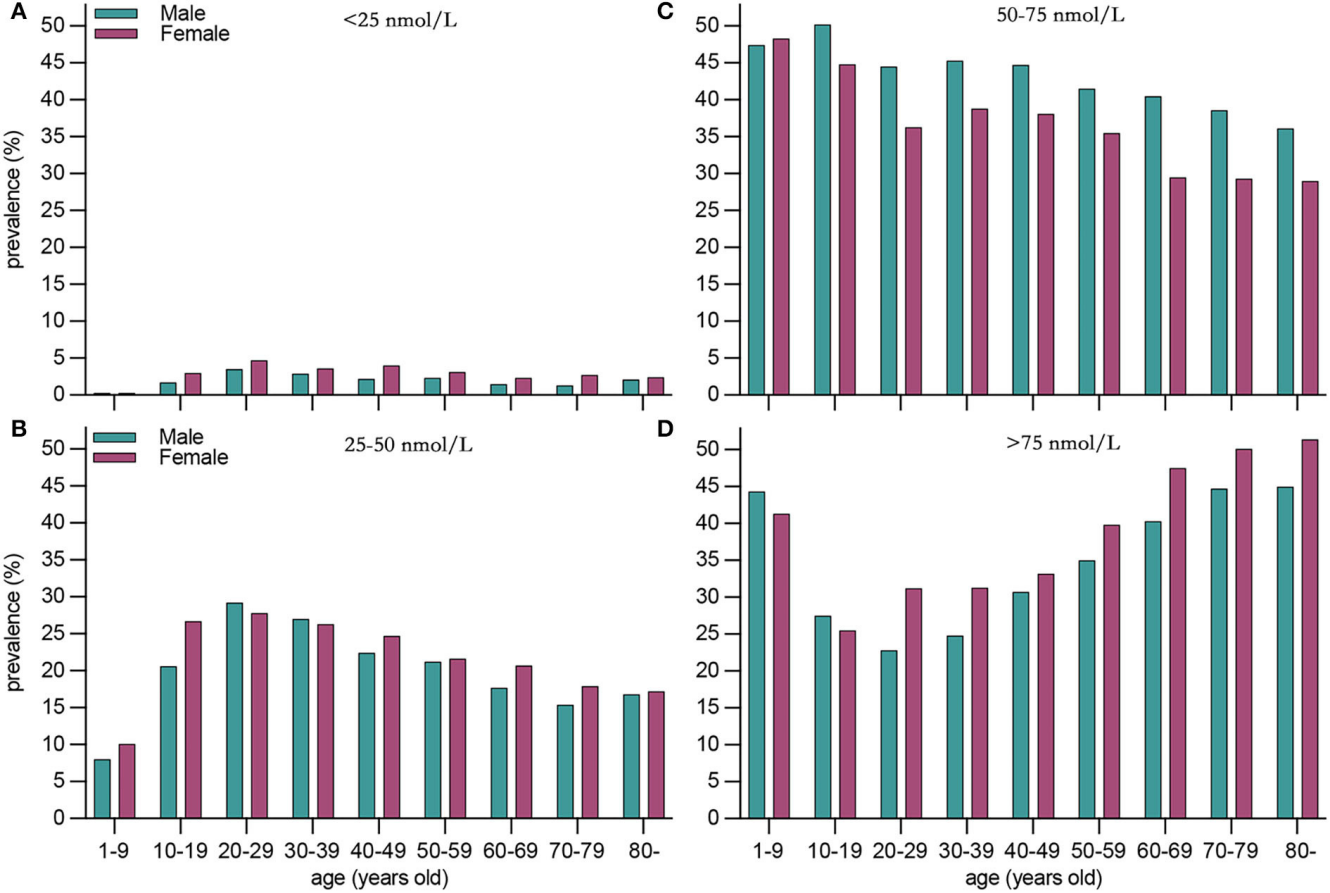
Weighted prevalence of serum 25(OH)D 75 nmol/L in the United States between 2001 and 2018 by sex and age. **(A)** <25 nmol/L, **(B)** <25–50 nmol/L, **(C)** 50–75 nmol/L, and **(D)** >75nmol/L.

**Figure 2:**
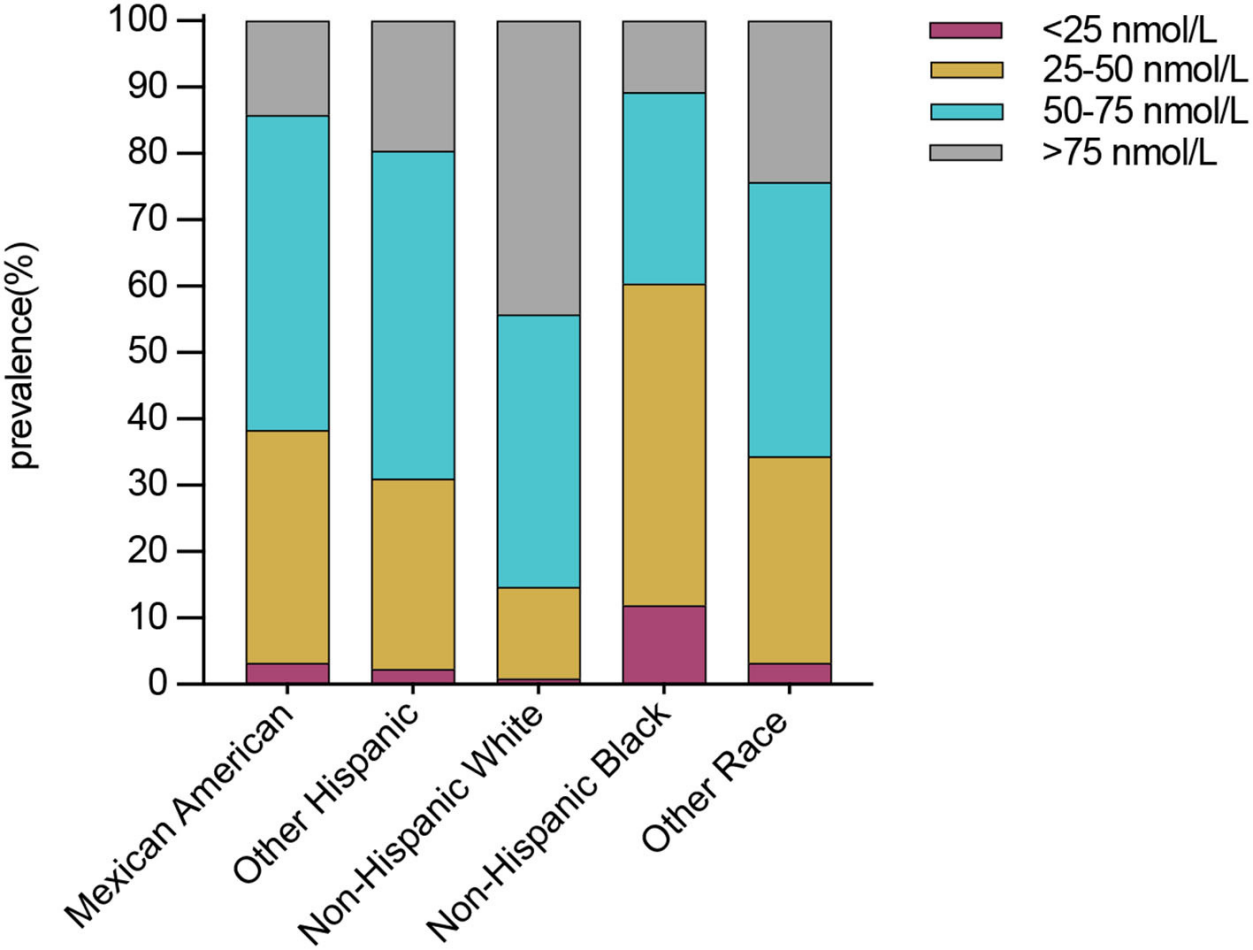
Weighted prevalence of serum 25(OH)D <25, 25–50, 50–75, and >75 nmol/L in Americans ages >1 year between 2001 and 2018 by ethnicity.

Compared with males, females presented a higher weighted proportion of serum 25(OH)D levels <25 nmol/L (3.1% vs. 2.1%, *P* <0.001) and 25–50 nmol/L (22.8% vs. 21.2%, *P* <0.001), but a lower weighted proportion of serum 25(OH)D levels 50–75 nmol/L (Table 2 and Figure 1). For ethnicity (Table 2 and Figure 2), the weighted proportion of serum 25(OH)D levels <25 and 25–50 nmol/L was lowest in non-Hispanic whites (0.9% and 13.8%, *P* <0.001) and highest in non-Hispanic blacks (11.9% and 48.5%, *P* <0.001). The weighted proportion of serum 25(OH)D levels of <25 nmol/L was lowest in aged 1–9 years (8.1% for boys and 10.1% for girls) and highest in ages between 20 and 29 years (29.1% for men and 28.1% for women). The same trend was also found in serum 25(OH)D levels of 25–50 nmol/L (Table 2 and Figure 1). Compared with summer, individuals in winter had a higher weighted proportion of serum 25(OH)D levels <25 nmol/L (4.1% vs. 1.6%, *P* <0.001) and 25–50 nmol/L (28.9% vs. 17.0% *P* <0.001), but a lower weighted proportion of serum 25(OH)D levels 50–75 nmol/L and >75 (Table 2 and Figure 1).

**Figure 3:**
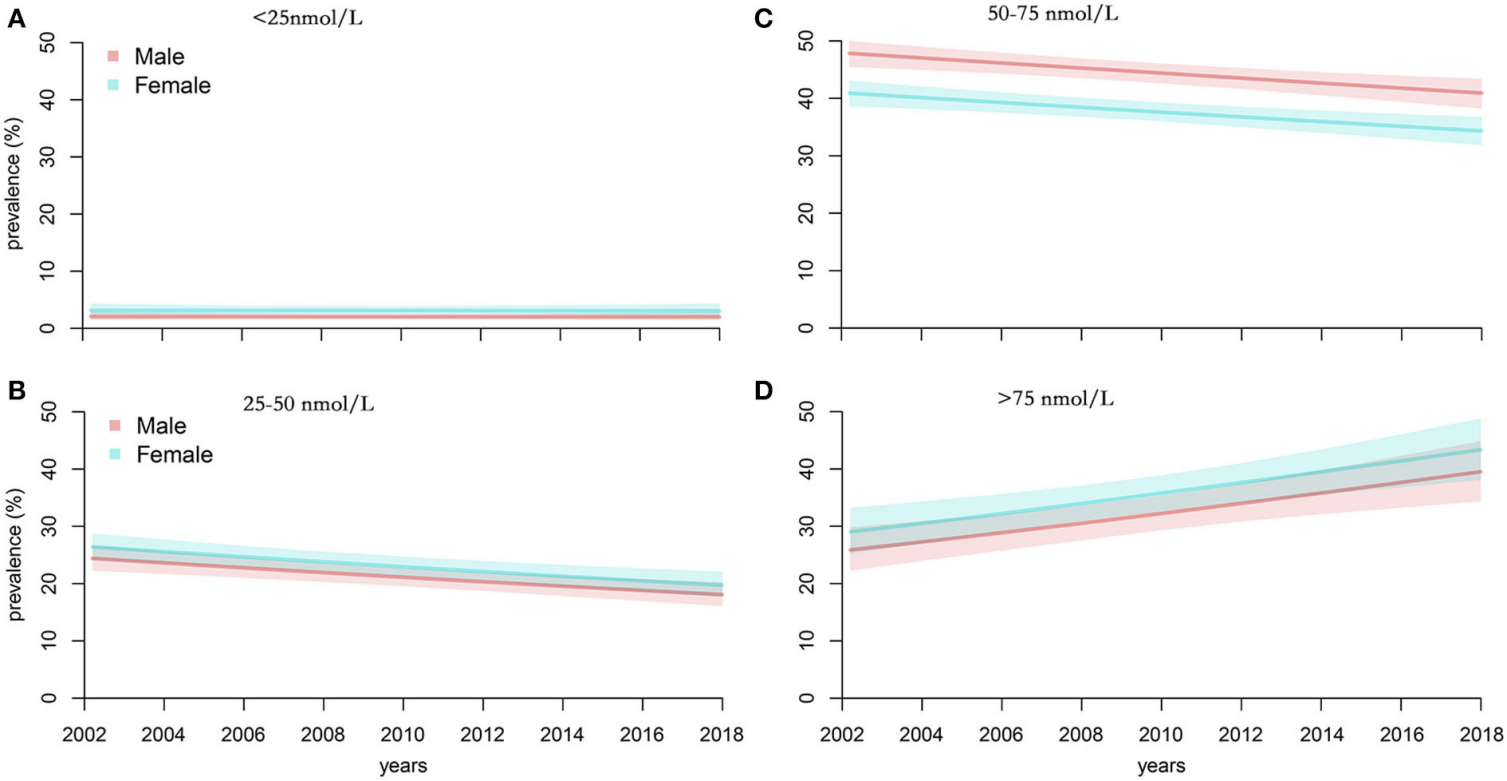
Trends of the prevalence of serum 25(OH)D 75 nmol/L in Americans aged >1 year from 2001 to 2018. **(A)** <25 nmol/L, **(B)** <25–50 nmol/L, **(C)** 50–75 nmol/L, and **(D)** >75nmol/L.

Table 3 and Figure 3 show the change trends of different vitamin D status in Americans from 2001 to 2018. We found a slight linear decrease in prevalence of moderate VDD [25 <25(OH)D <50 nmol/L] (coefficient = −0.847; *P* = 0.009) and VDI [50 <25(OH)D <75 nmol/L] (coefficient = −0.810; *P* = 0.014). We found a slight linear increase in vitamin D sufficient [25(OH)D >75 nmol/L] (coefficient = 1.693; *P* = 0.004). However, no trend change was found in severe VDD [25(OH)D <25 nmol/L] (coefficient = −0.037; *P* = 0.698). The same trend was also found after sex-specific analysis.

**Table 3:** Trends of the prevalence of serum 25(OH)D <25, 25–50, 50–75, and >75 nmol/L in the United States from 2001 to 2018.

	**Total**			**Males**			**Females**		
	**Sample size**	**Prevalence (%)**	**95% CI**	**Sample size**	**Prevalence (%)**	**95% CI**	**Sample size**	**Prevalence (%)**	**95% CI**
**25(OH)D** ** <25 nmol/l**									
Total	71,685	2.6%	(2.5%, 2.7%)	35,306	2.1%	(2.0%, 2.3%)	36,379	3.2%	(3.0%, 3.4%)
2001–2002	7,807	2.2%	(1.9%, 2.6%)	3,782	1.3%	(1.0%, 1.7%)	4,025	3.1%	(2.6%, 3.7%)
2003–2004	8,294	3.9%	(3.5%, 4.3%)	4,095	2.5%	(2.1%, 3.0%)	4,199	5.3%	(4.7%, 6.0%)
2005–2006	8,306	1.6%	(1.4%, 1.9%)	4,048	1.3%	(1.0%, 1.7%)	4,258	2.0%	(1.6%, 2.5%)
2007–2008	6,950	3.1%	(2.7%, 3.5%)	3,481	2.4%	(2.0%, 3.0%)	3,469	3.7%	(3.1%, 4.4%)
2009–2010	8,700	3.0%	(2.7%, 3.4%)	4,321	2.3%	(1.9%, 2.8%)	4,379	3.7%	(3.2%, 4.3%)
2011–2012	7,743	2.5%	(2.2%, 2.9%)	3,879	2.4%	(2.0%, 2.9%)	3,864	2.6%	(2.1%, 3.1%)
2013–2014	8,437	2.6%	(2.3%, 3.0%)	4,134	2.4%	(2.0%, 2.9%)	4,303	2.9%	(2.4%, 3.4%)
2015–2016	8,039	2.3%	(2.0%, 2.7%)	3,956	2.2%	(1.8%, 2.7%)	4,083	2.5%	(2.1%, 3.0%)
2017–2018	7,409	2.5%	(2.2%, 2.9%)	3,610	2.2%	(1.8%, 2.7%)	3,799	2.8%	(2.3%, 3.4%)
Trend		*P* = 0.698 β = −0.037			*P* = 0.196 β = 0.082			*P* = 2.259 β = −0.148	
**25** ** <25(OH)D** ** <50 nmol/l**									
Total	71,685	22.0%	(21.7%, 22.3%)	35,306	21.5%	(21.1%, 22.0%)	36,379	23.4%	(22.9%, 23.9%)
2001–2002	7,807	25.7%	(24.7%, 26.7%)	3,782	23.2%	(21.9%, 24.6%)	4,025	28.1%	(26.7%, 29.5%)
2003–2004	8,294	23.5%	(22.6%, 24.4%)	4,095	21.6%	(20.4%, 22.9%)	4,199	25.3%	(24.0%, 26.6%)
2005–2006	8,306	27.7%	(26.8%, 28.7%)	4,048	26.2%	(24.9%, 27.6%)	4,258	29.1%	(27.8%, 30.5%)
2007–2008	6,950	20.8%	(19.9%, 21.8%)	3,481	19.4%	(18.1%, 20.7%)	3,469	22.2%	(20.9%, 23.6%)
2009–2010	8,700	20.9%	(20.1%, 21.8%)	4,321	19.7%	(18.5%, 20.9%)	4,379	22.0%	(20.8%, 23.2%)
2011–2012	7,743	20.9%	(20.0%, 21.8%)	3,879	20.5%	(19.3%, 21.8%)	3,864	21.4%	(20.1%, 22.7%)
2013–2014	8,437	20.6%	(19.8%, 21.5%)	4,134	21.4%	(20.2%, 22.7%)	4,303	19.8%	(18.6%, 21.0%)
2015–2016	8,039	20.2%	(19.3%, 21.1%)	3,956	20.0%	(18.8%, 21.3%)	4,083	20.4%	(19.2%, 21.7%)
2017–2018	7,409	19.0%	(18.1%, 19.9%)	3,610	19.6%	(18.4%, 20.9%)	3,799	18.3%	(17.1%, 19.6%)
Trend		*P* = 0.009 β = −0.847			*P* = 0.108 β = −0.426			*P* =0.001 β = −1.222	
**50** ** <25(OH)D** ** <75 nmol/l**									
Total	71,685	40.9%	(40.5%, 41.3%)	35,306	44.7%	(44.2%, 45.3%)	36,379	37.8%	(37.3%, 38.3%)
2001–2002	7,807	45.1%	(44.0%, 46.2%)	3,782	48.0%	(46.4%, 49.6%)	4,025	42.3%	(40.8%, 43.8%)
2003–2004	8,294	42.4%	(41.3%, 43.5%)	4,095	45.3%	(43.8%, 46.8%)	4,199	39.7%	(38.2%, 41.2%)
2005–2006	8,306	45.7%	(44.6%, 46.8%)	4,048	47.9%	(46.4%, 49.4%)	4,258	43.6%	(42.1%, 45.1%)
2007–2008	6,950	39.9%	(38.8%, 41.1%)	3,481	44.7%	(43.1%, 46.4%)	3,469	35.3%	(33.7%, 36.9%)
2009–2010	8,700	39.6%	(38.6%, 40.6%)	4,321	44.3%	(42.8%, 45.8%)	4,379	35.1%	(33.7%, 36.5%)
2011–2012	7,743	37.6%	(36.5%, 38.7%)	3,879	40.3%	(38.8%, 41.8%)	3,864	35.0%	(33.5%, 36.5%)
2013–2014	8,437	40.7%	(39.7%, 41.8%)	4,134	44.6%	(43.1%, 46.1%)	4,303	37.0%	(35.6%, 38.5%)
2015–2016	8,039	39.1%	(38.0%, 40.2%)	3,956	43.2%	(41.7%, 44.8%)	4,083	35.1%	(33.7%, 36.6%)
2017–2018	7,409	38.5%	(37.4%, 39.6%)	3,610	42.1%	(40.5%, 43.7%)	3,799	35.2%	(33.7%, 36.7%)
Trend		*P* = 0.014 β= −0.810			*P* = 0.020 β = −0.682			*P* = 0.021 β = −0.928	
**25(OH)D** **>75 nmol/l**									
Total	71,685	34.5%	(33.2%, 34.9%)	35,306	31.7%	(31.2%, 32.2%)	36,379	35.6%	(35.1%, 36.1%)
2001–2002	7,807	27.0%	(26.0%, 28.0%)	3,782	27.5%	(26.1%, 29.0%)	4,025	26.5%	(25.2%, 27.9%)
2003–2004	8,294	30.2%	(29.2%, 31.2%)	4,095	30.7%	(29.3%, 32.1%)	4,199	29.7%	(28.3%, 31.1%)
2005–2006	8,306	25.0%	(24.1%, 26.0%)	4,048	24.6%	(23.3%, 26.0%)	4,258	25.3%	(24.0%, 26.6%)
2007–2008	6,950	36.2%	(35.1%, 37.3%)	3,481	33.5%	(32.0%, 35.1%)	3,469	38.8%	(37.2%, 40.4%)
2009–2010	8,700	36.4%	(35.4%, 37.4%)	4,321	33.7%	(32.3%, 35.1%)	4,379	39.1%	(37.7%, 40.6%)
2011–2012	7,743	39.0%	(37.9%, 40.1%)	3,879	36.9%	(35.4%, 38.4%)	3,864	41.1%	(39.6%, 42.7%)
2013–2014	8,437	36.1%	(35.1%, 37.1%)	4,134	31.6%	(30.2%, 33.0%)	4,303	40.4%	(38.9%, 41.9%)
2015–2016	8,039	38.4%	(37.3%, 39.5%)	3,956	34.6%	(33.1%, 36.1%)	4,083	42.1%	(40.6%, 43.6%)
2017–2018	7,409	40.0%	(38.9%, 41.1%)	3,610	36.0%	(34.5%, 37.6%)	3,799	43.7%	(42.1%, 45.3%)
Trend		*P* = 0.004 β = 1.693			*P* = 0.029 β = 1.052			*P* = 0.001 β = 2.308	

A linear trend test was used to identify trends in vitamin D status in Americans from 2001 to 2018. MEC weights were used. P-value <0.05 was considered statistically significant. β-regression coefficient, unit = % per cycle. MEC, Mobile Examination Center.

### Predictors of vitamin D deficiency

When using a cut-off of 25 nmol/L, the female was an independent predictor of VDD (OR 1.39 [95% CI 1.26–1.52]). In addition, race/ethnicity was a strong independent predictor of VDD. Compared with non-Hispanic white, Mexican Americans (OR 1.81 [95% CI 1.53–2.14]), other Hispanics (OR 1.73 [95% CI 1.38–2.16]), non-Hispanic black (OR [8.59 95% CI 7.48–9.86]), other race (OR 2.93 [95% CI 2.40–3.57]) have a higher risk of VDD. Compared with individuals aged <18 years old, VDD was common in individuals aged 18–44 years old (OR 3.06 [95% CI 2.53–3.71]), 45–65 years old (OR 2.51 [95% CI 2.00–3.14]), and >65 years old (OR 2.32 [95% CI 1.80–3.00]). Being assessed during winter was also an independent predictor of VDD (OR 2.06 [95% CI 1.89–2.26]). Moreover, lower PIR, lower education level, higher BMI, sun-protective behaviors (staying in the shade, wearing long sleeves), drinking, and lower milk consumption were also predictors of VDD. The details of predictors of VDD are listed in Table 4.

**Table 4:** The results of the predictors of vitamin D deficiency by weighted linear regression modeling.

	**25(OH)D level**<**25 nmol/L**			**25(OH)D level**<**50 nmol/L**		
	**OR**	**95 % CI**	***P***	**OR**	**95 % CI**	***P***
Sex (vs. men)	[Reference]			[Reference]		
Women	1.39	(1.26, 1.52)	<0.01	1.17	(1.13, 1.22)	<0.01
Race/ethnicity (vs. non-Hispanic white)				[Reference]		
Mexican American	1.81	(1.53, 2.14)	<0.01	2.55	(2.41, 2.69)	<0.01
Other Hispanics	1.73	(1.38, 2.16)	<0.01	2.12	(1.98, 2.28)	<0.01
Non-Hispanic black	8.59	(7.48, 9.86)	<0.01	7.16	(6.80, 7.55)	<0.01
Other race	2.93	(2.40, 3.57)	<0.01	3.06	(2.86, 3.29)	<0.01
Age (vs. <18)	[Reference]			[Reference]		
18–44	3.06	(2.53, 3.71)	<0.01	2.36	(1.86, 2.28)	<0.01
45–65	2.51	(2.00, 3.14)	<0.01	1.71	(0.88, 1.18)	<0.01
>65	2.32	(1.80, 3.00)	<0.01	1.45	(0.51, 0.75)	<0.01
Education **(**vs. high school degree and below**)**	[Reference]			[Reference]		
College degree and above	0.89	(0.80, 0.99)	0.03	0.88	(0.85, 0.93)	<0.01
PIR (vs. <1.3)	[Reference]			[Reference]		
1.3–3.5	0.89	(0.80, 0.98)	0.02	0.93	(0.89, 0.97)	<0.01
>3.5	0.68	(0.60, 0.77)	<0.01	0.74	(0.70, 0.78)	<0.01
Sun-protective behaviors						
Staying in the shade (vs. Rare)	[Reference]			[Reference]		
Sometimes	1.15	(0.94, 1.40)	0.17	1.12	(1.02, 1.22)	0.01
Frequent	1.44	(1.19, 1.75)	<0.01	1.46	(1.34, 1.60)	<0.01
Wearing long sleeves (vs. Rare)	[Reference]			[Reference]		
Sometimes	1.05	(0.88, 1.26)	0.56	1.10	(1.02, 1.21)	0.01
Frequent	1.43	(1.16, 1.77)	<0.01	1.17	(1.05, 1.30)	<0.01
Using sunscreen (vs. Rare)	[Reference]			[Reference]		
Sometimes	0.71	(0.57, 0.89)	<0.01	0.77	(0.70, 0.84)	<0.01
Frequent	0.65	(0.51, 0.82)	<0.01	0.74	(0.67, 0.81)	<0.01
Season (vs. Summer)	[Reference]			[Reference]		
Winter	2.08	(1.89, 2.26)	<0.01	0.56	(0.54, 0.59)	<0.01
Had at least 12 alcohol drinks/1 year? (vs. yes)	[Reference]			[Reference]		
No	0.83	(0.74, 0.93)	<0.01	0.91	(0.87, 0.96)	<0.01
Smoke at least 100 cigarettes in life (vs. yes)	[Reference]			[Reference]		
No	1.02	(0.91, 1.15)	0.69	1.17	(1.10, 1.24)	<0.01
Physical activity (vs. Rare)	[Reference]			[Reference]		
Sometimes	0.77	(0.35, 1.69)	0.52	0.84	(0.62, 1.14)	0.26
Regular	0.57	(0.27, 1.21)	0.14	0.58	(0.43, 0.76)	<0.01
BMI (vs. <18.5)	[Reference]			[Reference]		
18.5–25	2.21	(1.82, 2.69)	<0.01	2.21	(1.82, 2.69)	<0.01
>25	3.03	(2.49, 3.68)	<0.01	3.03	(2.49, 3.68)	<0.01
Milk consumption (vs. Rare)	[Reference]			[Reference]		
Sometimes	0.77	(0.69, 0.88)	<0.01	0.84	(0.79, 0.90)	<0.01
Regular	0.44	(0.38, 0.50)	<0.01	0.65	(0.61, 0.69)	<0.01

MEC weights were used. Adjusted for age, gender, race/ethnicity, BMI, PIR, drinking behavior, sun-protective behaviors, season, drinking behavior, smoking behavior, physical activity, and milk consumption. MEC, mobile examination center; PIR, poverty income ratio; BMI, body mass index.

## Discussion

To our knowledge, this study is the latest and most comprehensive estimate of the vitamin D status of the American population using the available data from NHANES (2001–2018). The main findings of our study were that the prevalence of serum 25(OH)D <25, 25–50, 50–75, and >75 nmol/L was 2.6%, 22.0%, 40.9%, and 34.5% in Americans aged >1 year old between 2001 and 2018. Age, sex, ethnicity, season, sun-protective behaviors, lower BMI, lower SES, drinking, and lower milk consumption were predictors of VDD.

Our results also showed that the prevalence of severe VDD [25(OH)D <25 nmol/L] had not improved significantly, and the moderate deficiency [25 <25(OH)D <50 nmol/L], insufficiency [50 <25(OH)D <75 nmol/L] had a mild improvement in the United States in recent years. Several potential mechanisms could explain it. First, with the increased health awareness of the general population in the United States in recent years, there has been a surge in serum 25(OH)D testing and diagnoses of VDD (26, 27). Second, healthcare providers are increasingly recommending higher doses of vitamin D supplements than previously (28), and people have a 10-fold increase in spending on vitamin D supplements between 2001 and 2009 (29). Schleicher et al. (30) estimated the use of vitamin D supplements in Americans based on NHANES and showed a significant increase in the use of vitamin D supplements in the general population. In 2003–2004, only 0.45% of adults (>20 years) used ≥1,000 IU/d vitamin D-containing supplements. The percentage increased to 16.12% in 2013–2014. Furthermore, more and more foods in the U.S. are being fortified with vitamin D (31). Nevertheless, vitamin D status in the U.S. population has not improved significantly in recent years, which may lead to adverse consequences (32–34). More action is needed by the government and medical providers to provide effective prevention and treatment strategies for the disease.

Vitamin D deficiency is often associated with insufficient sunlight, as humans obtain most of their vitamin D through UVB radiation. A previous study reported a negative association between the serum level of 25(OH)D and distance from the equator (35). The United States is a high-latitude country where lower UVB radiation may cause a higher incidence of VDD than in countries with ample sunshine (36). However, our results show that the prevalence of VDD in Americans was lower than in some low-latitude countries (13, 37), where the population has a higher direct UV-B exposure. A large meta-analysis conducted by Mogire et al. (13) reported that 18·46% and 34·22% of Africans have VDD when using a cut-off of 30 and 50 nmol/L, respectively. Pereira-Santos et al. (37) reported that 28.16% of Brazilians suffered from VDD when using a cut-off of 50 nmol/L, higher than the result of our study (24.6%). It could mean that other factors besides latitude or sun exposure may affect vitamin D status, like race/ethnicity, cultural practices, and other factors (38, 39). For instance, in the United States, food supplementation and fortification are common sources of vitamin D (40), but they are unavailable in many African and South American countries (13). Cultural practices also have an impact on vitamin D status. For example, Middle Easterners often wear veils (covering skin from sunlight), which could lead to a higher prevalence of VDD (41). Our results also show a lower prevalence of VDD [serum 25(OH)D <50 nmol/L] in the United States than in Europe. A nationally representative study in Europe showed that 40.4% of these populations had serum 25(OH)D <50 nmol/L (42), whereas we found a prevalence of 24.6% in the United States.

The prevalence of VDD varied greatly based on the different cut-offs, race/ethnicity, sex, age, tested season, and so on. The prevalence of serum 25(OH)D <25 and 25–50 nmol/L is highest among non-Hispanic blacks and lowest among non-Hispanic whites, the same as the previous studies (43–45). This relationship persisted after controlling for other variables. Dark-skinned people, especially non-Hispanic blacks, have pigment melanin in their skin that can absorb sunlight, which decreases the synthesis of vitamin D (46, 47). In addition, studies have shown that lower serum 25(OH)D levels in African-Americans may be associated with obesity (48, 49). However, an article showed that non-Hispanic blacks with serum 25(OH)D below the cut-off typically lack the accompanying characteristic alterations (38). A previous community-based study showed that black Americans have similarly bioavailable 25(OH)D concentrations to white Americans, although they had lower levels of a total of 25(OH)D and a vitamin D receptor (VDR) (38). Using the same 25(OH)D cut-off to define VDD for diverse populations may not be appropriate and need further investigation.

In addition to race, the present study shows that the prevalence of serum 25(OH)D <25 and <50 nmol/L is highest in people aged 20–29 in America. After controlling for variables, our study showed that being 18–44 was also a predictor of VDD. The age-specific trends in VDD prevalence varied from study to study, but most results showed a higher prevalence in young adulthood (19, 21, 50). One explanation is that older people are more likely to use vitamin D supplements than younger people (51). Moreover, a cross-sectional study conducted in rural America shows that younger people are more likely to use sunscreen than older people (52).

In this present study, PIR and education level are considered to be the two main measures of SES (53), which have been identified as independent predictors for VDD. Individuals with low SES may have low disease awareness and may not like consuming foods high in vitamin D, such as fish and milk (54). The literature shows that low SES (low income) may limit the potential to purchase more expensive and vitamin D-rich foods (e.g., sea fish, fish oil, fortified foods, and eggs) and do regular physical examinations to find VDD timely (55). For instance, European women with lower SES are less likely to use vitamin D supplements (56, 57). Lin et al. reported that low SES was associated with an elevated risk of VDD in women of childbearing age (58).

Other studies have widely discussed the potential mechanisms for these connections between BMI, alcohol consumption, sun-protective behaviors, and milk consumption with vitamin D status (59–62). Notably, the relationship between sunscreen use and vitamin D status is contrary to previous studies (63, 64). It may be due to the limitations of the questionnaire, as regular sunscreen users tend to be more exposed to sunlight.

Our study has several obvious advantages. On the one hand, this is a large sample analysis based on the NHANES survey. The sample of this study adopted multi-layer random sampling, with high reliability and standardization of data, which can represent the general population of the United States. On the other hand, we used the currently available vitamin D data from NHANES 2001–2018 and used different cut-offs to analyze vitamin D status in Americans. Some limitations should be acknowledged. First, since this is a cross-sectional study, no causal relationship between predictors and VDD can be inferred. Second, for technical reasons, serum 25(OH)D concentrations were measured by RIA kit in 2001–2006 and LC-MS/MS method in 2007–2018, which may lead to instability of results. However, vitamin D data measured by RIA kit date were adjusted and converted to equivalent measurements by LC-MS/MS methods. Third, because vitamin D status is influenced by factors, such as season and vitamin supplement use, we were unable to assess these specific factors due to the limited information available from NHANES. Fourth, since the United States is a country with vast geographical and latitudinal differences, we could not assess them because of the limited information available from NHANES. Further studies are needed to explore it in the future.

## Conclusion

Vitamin D deficiency is still prevalent in the United States, especially in non-Hispanic blacks, women, individuals aged 20–29 years, and during the season of winter. Individuals, healthcare providers, and policymakers should take public health measures to develop and implement prevention strategies for VDD.

## Data availability statement

The datasets presented in this study can be found in online repositories. The names of the repository/repositories and accession number(s) can be found below: https://www.cdc.gov/nchs/nhanes/.

## Author contributions

AC, PX, FZ, LZ, and JZ: conceptualization, project administration, and visualization. AC, PX, YM, and LZ: data curation. AC, PX, YM, ZF, FZ, LZ, and JZ: formal analysis, investigation, and writing—review and editing. AC, PX, YM, and LZ: methodology. AC, PX, and LZ: software.

## Conflict of interest

The authors declare that the research was conducted in the absence of any commercial or financial relationships that could be construed as a potential conflict of interest.

## Publisher's note

All claims expressed in this article are solely those of the authors and do not necessarily represent those of their affiliated organizations, or those of the publisher, the editors and the reviewers. Any product that may be evaluated in this article, or claim that may be made by its manufacturer, is not guaranteed or endorsed by the publisher.
