# Supplementary material for: Prevalence, trend, and predictor analyses of vitamin D deficiency in the US population, 2001–2018
Source: Front Nutr. 2022 Oct 3;9:965376. doi: 10.3389/fnut.2022.965376 (PMC9573946; doi:10.3389/fnut.2022.965376)
Supplement: Supplementary file 1 [file Table_1.DOCX]

**appendix 1**

Serum 25-hydroxyvitamin D (25(OH)D) data from NHANES 2001-2006 have been converted by using regression to equivalent 25(OH)D measurements from a standardized liquid chromatography-tandem mass spectrometry (LC-MS/MS) method

2001-2002: LC-MS/MSequivalent = 6.43435 + 0.95212*RIAoriginal;

2003-2004: LC-MS/MSequivalent = 1.72786 + 0.98284*RIAoriginal;

2005-2006: LC-MS/MSequivalent = 8.36753 + 0.97012*RIAoriginal)
